# Lancaster Town: Its Mayor and Its Hospital

**Published:** 1887-01-15

**Authors:** 


### Lancaster Town: its Mayor and its Hospital.

By our Special Commissioner.

Mr. Thomas Storey, the Mayor of Lancaster, in a recent speech at the Town Hall, alluded to the Lancaster Infirmary, the inconvenience of its buildings, and the state of dirt and mismanagement which appeared to him to be prevalent there when he visited the institution some years ago; adding, however, that he believed its present condition to be "something near what it ought to be." Thereupon the Senior Medical Officer, Dr. John Harker, wrote to the local press, defending the past and present management. In the course of this letter, however, Dr. Harker admitted the inconvenience of the existing buildings, and offered to start a subscription towards a new infirmary, with a gift of £100 from his own purse. Under these circumstances, a description of the present institution by an unprejudiced observer may be of interest to our readers as well as helpful to the people of Lancaster.

**The Buildings.** The Lancashire Infirmary Buildings stand on a small quadrangular space, surrounded by streets on every side. They consist of a main building, formerly a private dwelling-house, a small left wing used as a scullery, and a right wing, erected at right angles to the original building. Two walls complete the enclosure, and, in the corner formed by their junction, is placed the mortuary. The centre building contains: kitchens, larders, etc., in the basement; the board-room, officers' sitting-rooms, and dispensary on the ground floor; and in the upper floors two female wards, roughly calculated as 24 feet long, 18 wide and 11 in height, and 32 feet by 18 by 12, on the first and second floor respectively; with a small operating-room, a special room for ovarian and other cases, and bedrooms for the staff. The kitchens, etc., though ample for their original purpose, are not adequate to the requirements of the thirty or more resident patients and officials; and the dispensary is also deficient in waiting-room space, part of it being lumbered up with packages, etc., and used as a drug-store. The board- and sitting-rooms, however, are fairly good, but the staircase leading to the female wards is both narrow and awkward. The operating-room, on the first-floor, is small, partly lighted from the top, and is also used as a bath and store-room. The only convenience for female patients is on the upper storey, opening directly on the lobby outside the ward door. It is of old-fashioned character, and reveals its presence by an unmistakable odour. There is no lavatory or bath-room on this floor, the patients using a portable bath at the bedside, when necessary. Both wards were neatly painted, and the bedding was clean, plentiful, and good. There were six patients in the lower, and seven in the upper ward at the time of my visit.

**The Wards.** The male wards in the right wing are approached by a lobby continuous with the first-floor female lobby, and the staircase used by patients on their way to the wards is narrow, with a very awkward turn. It is found necessary to remove the balusters in order to get a stretcher up these stairs when serious accidents are admitted. The two male wards open one out of the other, and consist really of one long room with a partition wall and door in the centre. The rooms thus divided are about 30 feet long by 18 feet, and 10 feet in height, and have in th m eight and seven beds respectively. At the end of the inner room there is a stove, and a room about 8 feet square, with a bath and closet, which was also offensive, the window being closed, and the door opening directly into the ward. The bath in this room depends upon the laundry copper for its supply, so that when the laundry fire is out there is no hot water. The beds in the male wards are too close together, and there is neither floor space nor cubic space for more than half the number of beds. This remark applies quite as strongly to the female wards. The ventilation of the female wards is not satisfactory. We were not surprised to learn that the wards are often very stuffy when all the beds are occupied; and as there is no day-room for convalescents, the patients have no other resource but to occupy the wards when out of bed. The men's wards having windows on both sides, have a better chance as regards ventilation, but these old-fashioned casements can hardly be opened without exposing the patients to draughts. With such cramped accommodation there can be no systematic separation of surgical and medical cases, and the patients are consequently treated side by side without any attempt at classification.

**Nurses and Patients.** The nursing staff consists of a trained nurse and two probationers; one of the latter does the night nursing, the other two being on duty in the day-time. There are also four maids for the house-work. The dinner was about to be served at the close of our visit, and was very nice and appetising, the carving being done by the matron, who is thus able to assure herself that every patient is suitably and properly helped. I saw a printed dietary table which seemed both liberal and varied. There were twenty-six patients under treatment, thirteen of either sex, and five vacant beds. There is often great difficulty in providing accommodation for all those who need it, and on occasions of sudden accidents some of the patients approaching convalescence have to be sent out in order to make beds for the most urgent cases. Upwards of 300 in-patients have been under care during the past year, and it is evident that the Infirmary is hardly large enough for the increasing wants of the town, while it will also be seen from these notes that in its equipment as a hospital it does not quite come up to the modern idea of such an institution.

**Build a Hospital.** Much good work is being done, but the evident overcrowding and want of space place the nursing staff at great disadvantage, whilst they increase the risks to the patients in cases requiring operation. We learn from the Secretary that the building of a new Infirmary is in contemplation, and that negotiations for the purchase of an admirable site are in progress. Let us hope that all classes of the community in Lancaster and the neighbourhood will come forward at this critical era in the history of the Infirmary, and contribute such a sum as will enable the Committee to build a Hospital equal in every way to the requirements of the district.

